# Myocardial histology and outcome after cardiopulmonary bypass of neonatal piglets

**DOI:** 10.1186/s13019-015-0380-0

**Published:** 2015-11-20

**Authors:** Theodor Tirilomis, Marc Bensch, Regina Waldmann-Beushausen, Friedrich A. Schoendube

**Affiliations:** Department for Thoracic, Cardiac, and Vascular Surgery, University of Goettingen, Robert-Koch-Str. 40, 37075 Goettingen, Germany

**Keywords:** Myocardial tissue, Cardiopulmonary bypass, Neonatal, Hypothermia, Ischemia

## Abstract

**Background:**

Early after neonatal cardiac surgery hemodynamic dysfunction may be evident. However, still is not clear if dysfunction and outcome is related to visible myocardial alterations. The aim of the present study was the histological analysis of myocardial tissue of neonatal piglets after cardiopulmonary bypass (CPB) and cardioplegic arrest.

**Methods:**

Neonatal piglets (younger than 7 days) were connected to CPB for 180 min, including 90 min of cardioplegic heart arrest at 32 °C. After termination of CPB the piglets were observed up to 6 h. During this observational period animals did not receive any inotropic support. Some piglets died within this period and formed the non-survivors group (CPB-NS group) and the remaining animals formed the CPB-6 h group. Myocardial biopsies (stained with H&E) were scored from 0 to 3 regarding histological alterations. Then, the histological data were evaluated and compared to the probes of animals handled comparable to previous piglets but without CPB (non-CPB group; *n* = 3) and to sibling piglets without specific treatment (control; *n* = 5).

**Results:**

In the first hours after CPB six piglets out of 10 died (median 3.3 h). The animals of CPB-6 h group (*n* = 4) were sacrificed at the end of experiments (6 h after CPB). Although the myocardial histological score of CPB-6 h group and CPB-NS group were higher than non-CPB group (2.0 ± 0.8, 1.5 ± 0.9, and 0.8 ± 0.3 respectively), these differences were statistically not significant. But compared to control animals (score 0.3 ± 0.5) the scores of CPB-6 h and CPB-NS groups were significantly higher (*p* < 0.05). Between the left and the right ventricular tissue there were no significant differences.

**Conclusions:**

Myocardial tissue alterations in newborn piglets are related to the surgical trauma and potentiated by cardiopulmonary bypass and ischemia. However, outcome is not related to the degree of tissue alteration.

## Background

Correction of even complex congenital defects in newborns has excellent results. Nevertheless, in the first hours after repair hemodynamic instability may appear. This period may be attributed to myocardial dysfunction in the meaning of myocardial stunning, which is triggered by many mechanisms [1]. The majority of these mechanisms are perfusion-related and they are mostly identified in studies of adults with coronary artery disease and impaired myocardial perfusion prior to surgery. But conditions with low-flow ischemia or ischemia/reperfusion injury may appear also in newborns with congenital heart diseases.

One of the questions arising in this context is regarding myocardial histology: is the possible postoperative myocardial dysfunction associated with visible alterations of myocardial tissue and if yes, is outcome related to these alterartions? The aim of this study was to examine heart biopsies of neonatal piglets after cardiopulmonary bypass (CPB) and cardioplegic arrest, regarding myocardial tissue alterations and finally, to compare these possible changes to the histological findings of control piglets.

## Methods

The experimental protocol was approved by the Animal Care and Use Committees of the University of Goettingen and of the Government of the District of Braunschweig, Germany. All animals were handled according to the Federal Laws and in compliance with the European Convention on Animal Care. Anesthesia was induced with intramuscular azaperone and ketamine and maintained with intravenous ketamine and pentobarbital. Mechanical ventilation after tracheotomy was with air, oxygen, and isoflurane mixture. Exposure of the heart was achieved through median sternotomy. After systemic anticoagulation with heparin (300 U/kg) piglets were connected to cardiopulmonary bypass via cannulation of the ascending aorta and the right atrium. CPB was initiated with a flow rate of 2.5 l/min/m^2^. The activated clotting time was maintained at a value >400 s throughout perfusion time.

Initially, 10 neonatal piglets (younger than 7 days) were connected to CPB. After perfusion of 30 min and cooling to 32 °C core temperature a cardioplegic heart arrest has initiated for 90 min (crystalloid *Bretschneider’s* solution (Custodiol HTK, Köhler Chemie, Bensheim, Germany), 30 ml/kg over 7 min). Thereafter, the aortic clamp was released and a reperfusion time of 60 min with re-warming to 37 °C did follow. After a complete CPB time of 180 min the piglets were weaned off bypass and the cannulae were removed. Anticoagulation was reversed by administration of protamine sulfate.

During the postoperative course piglets did not receive any inotropic support or vasopressors. After an observational period of 6 h survived animals were sacrificed (CPB-6 h group). Some animals died during this period and formed the non-survivors group (CPB-NS group).

The hearts were explanted and myocardial biopsies were taken. The biopsies were kept in formalin 10% for 24 to 36 h. Thereafter, specimens were washed in aqua bidest for 1 h and cleaned with alcohol. After chloroform fixation, they were embedded in paraffin (Kendall, Tyco Healthcare Group KG, Mansfield, USA). Then, sections were cut at 1 μm with a microtome (Leica SM 2000R, Nussloch, Germany), washed (45 °C water), and dried at room temperature for night. Thereafter, slides were treated with xylol/ethanol and finally, stained with hematoxylin and eosin.

Histological examination of specimens was performed by 25-fold, 100-fold, and 250-fold magnification (microscope BH2, Olympus Europa GmbH, Hamburg, Germany). The severity of myocardial tissue damage was scored regarding myocardial edema, leukostasis, cell necrosis, and focal bleeding by a four-grade system described by Qing et al. [2], as follows: grade 0 shows normal morphology; grade 1 slight alterations; grade 2 moderate alterations; and grade 3: severe alterations.

The data of tissue probes were evaluated and compared to the probes of animals handled comparable to previous piglets but without CPB (non-CPB group; *n* = 3) and to sibling piglets without specific treatment (control; *n* = 5). The non-CPB animals underwent also tracheostomy, received mechanical ventilation, and after sternotomy catheters were placed into the vessels and into the heart. These animals were observed for the same time interval of 3 h in the meaning of time simulation for CPB duration and after this CPB-simulation time of 3 h for additional 6 h (“post-CPB time”).

The histological scoring was performed blinded to animal group. Statistical analysis was performed with Statistica 10 software (StatSoft (Europe) GmbH, Hamburg, Germany). Data were expressed as mean ± standard deviation. Data analysis was performed using Student’s *t*-test. Statistical significance was assumed for *p* < 0.05.

## Results

**Fig. 1:**
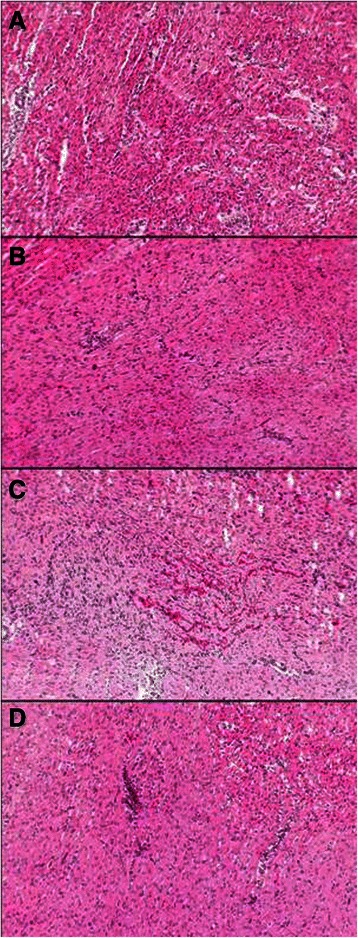
Hematoxylin and Eosin (H&E) staining of myocardial tissue (25× magnification) after cardiopulmonary bypass. **a** presents nearly normal tissue (grade 0), **b** shows grade 1 with beginning endothelial damage, **c** presents grade 2 with mild infiltration of inflammatory cells and focal bleeding, and **d** demonstrates grade 3 severe damage and infiltration of granulocytes

Myocardial alterations of all histological grades were found in treated groups (Fig. 1).

**Fig. 2:**
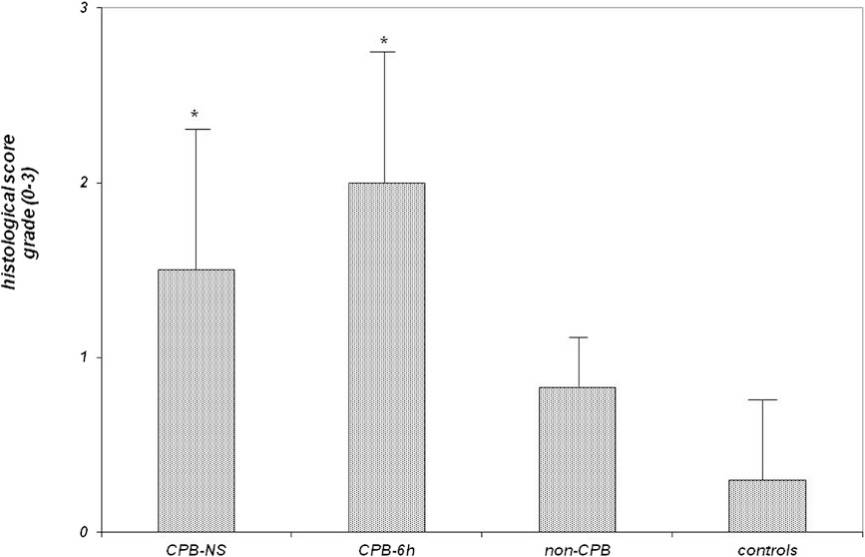
Presentation of the average scores in myocardial tissue of neonatal piglets (left ventricle). *p < 0.05 vs. controls

Although six piglets died within the first hours after CPB (median 3.3 h), the scores between the survived and non-survived piglets were not statistically different: CPB-6 h vs. CPB-NS: 2.0 ± 0.8 vs. 1.5 ± 0.9, *p* = 0.378 (Fig. 2). Also in comparison to the non-CPB group the myocardial histological scores of CPB-6 h group and CPB-NS group were higher but the differences were not statistically significant: CPB-6 h vs. non-CPB: 2.0 ± 0.8 vs. 0.8 ± 0.3, *p* = 0.068; CPB-NS vs. non-CPB: 1.5 ± 0.9 vs. 0.8 ± 0.3, *p* = 0.234. Only if compared to control animals (score 0.3 ± 0.5) the scores of CPB-6 h and CPB-NS groups were significantly higher (*p* = 0.005 and *p* = 0.019, respectively).

Between the left-ventricular and the right-ventricular tissue there were no significant differences (Table 1).

**Table 1:** Scoring results of left-ventricular (LV) and right-ventricular (RV) neonatal myocardial tissue after cardiopulmonary bypass and cardiac arrest (protected by crystalloid cargioplegia)

	LV	RV
CPB-NS	1.50 ± 0.84	1.33 ± 0.82
CPB-6 h	2.00 ± 0.82	1.88 ± 0.85
Non-CPB	0.83 ± 0.3	0.5 ± 0
Control	0.3 ± 0.45	0.3 ± 0.27

There are no statistically significant differences between the left and the right ventricle

## Discussion

Our results demonstrate that after surgery, even without cardiopulmonary bypass and without myocardial ischemia alterations of myocardial tissue are present. Although the alterations are not statistically significant they are evident. The additional cardiac procedure on CPB potentiates the myocardial alterations. Nevertheless, these results due not identify the cause of myocardial alterations. Many factors could be responsible: (i) myocardial ischemia, (ii) reperfusion injury, (iii) hypothermia, and (iv) cardiopulmonary bypass and its consequences (e.g., systemic inflammatory response).

Whether the neonatal myocardium is more vulnerable or more tolerant to ischemic injury and reperfusion injury remains controversial. Karimi et al. found in a neonatal animal model greater myocardial apoptosis [3] and they concluded that neonatal myocardium in the early postoperative period is more vulnerable to ischemia/reperfusion then mature myocardium. Although studies of the same group and other groups support this conclusion [4, 5], other studies support in contrast the hypothesis that neonatal myocardium is more resistant to ischemia/reperfusion [6, 7].

Hypothermia seems to be less responsible due to its well recognized cytoprotective effects [8]. Probably the myocardial alterations would be more prominent if we had performed the experiments in normothermia but we did apply hypothermia in our model because we wanted to reproduce our daily practice and we repair congenital heart defects in neonates on hypothermic CPB.

Cardiopulmonary bypass induces the release of inflammatory mediators and subsequently of the inflammatory cells [9]. The effects of the inflammatory response are not limited to the heart. The presented histological results confirm more likely our hemodynamic results, published previously [10]: the outcome of neonatal piglets is related to postoperative hypotension caused by vasoplegia.

Important limitations of our study are the small size of the cohort and the fact, that the piglets had normal hearts. Abnormal hearts may have myocardial alterations prior to surgery and therefore probably unexplained and unpredictable reaction on surgical procedure, CPB, and ischemia/reperfusion may result.

## Conclusions

The present study revealed significant myocardial tissue alterations after cardiac arrest on cardiopulmonary bypass in newborn piglets. These alterations are primarily related to surgical trauma and then potentiated by the myocardial ischemia. Additionally, the data show that outcome is not related to degree of tissue alteration.
